# ECHS1 as a Lipid Metabolism Biomarker for Pediatric Focal Segmental Glomerulosclerosis

**DOI:** 10.1371/journal.pone.0319049

**Published:** 2025-03-10

**Authors:** Chao He, Wei Peng, Sheng Li, Can Xu, Xiuping Chen, Yuanhan Qin

**Affiliations:** 1 Department of Pediatrics, The First Affiliated Hospital of Guangxi Medical University; 2 The First Affiliated Hospital, Department of Pediatrics, Hengyang Medical School, University of South China; 3 Department of Pediatrics, People’s Hospital of Ningxiang City; 4 The First Affiliated Hospital, Department of Cardiology, Institute of Cardiovascular Disease, Hengyang Medical School, University of South China; Sultan Qaboos University College of Medicine and Health Science, OMAN

## Abstract

Focal segmental glomerulosclerosis (FSGS) is a common cause of nephrotic syndrome and often leads to end-stage renal disease. However, the underlying pathophysiological mechanisms that contribute to disease progression require further investigation to establish appropriate therapeutic targets and biomarkers. This study aimed to clarify the molecular mechanisms underlying FSGS by focusing on differentially expressed genes (DEGs) and lipid metabolism-related genes (LMRGs). We utilized the GSE69814, GSE129973, and GSE121233 datasets, which comprise glomerular transcriptomes from patients with FSGS, minimal change disease (MCD), and unaffected kidney tissues. We identified 2,459 DEGs from the GSE69814 dataset and 982 DEGs from the GSE129973 dataset. These DEGs intersected 1,450 LMRGs, resulting in 56 differentially expressed LMRGs (DELMRGs). Enrichment analysis revealed that these DELMRGs were primarily involved in fatty acid metabolic processes; localized in microbodies, peroxisomes, and mitochondrial matrices; and exhibited oxidoreductase activity. Protein-protein interaction networks were constructed using Cytoscape, and five hub DELMRGs (enoyl-CoA hydratase, short chain 1 [*ECHS1*]*, EHHADH, IDH1, SUCLG1*, and *ALDH3A2*) were identified using multiple algorithms. We assessed the diagnostic performance using receiver operating characteristic curves and expression levels from the GSE121233 dataset, and found that *ECHS1* and *ALDH3A2* showed strong diagnostic potential. Immunohistochemical verification of clinical specimens from children confirmed significant expression of *ECHS1* in FSGS compared with that in normal and MCD tissues. This study highlights *ECHS1* as a potential biomarker for pediatric FSGS, suggesting a potential role in early diagnosis or personalized treatment, offering insights into its pathogenesis and paving the way for targeted therapeutic strategies.

## Introduction

Idiopathic nephrotic syndrome (INS) is the most common disorder affecting glomerular function in children [1]. Although more than 80% of INS cases are sensitive to glucocorticoid therapy, 10-20% of patients with INS still exhibit poor treatment response and are categorized them as refractory nephrotic syndrome (RNS) in children [2]. The incidence of RNS has increased in both children and adults, especially in certain groups, such as patients with diabetes and immune-mediated diseases, where RNS is more prevalent [3,4]. RNS affects the patient’s quality of life and can lead to severe complications, such as end-stage renal disease (ESRD) and cardiovascular diseases, making in-depth research clinically significant [2]. The incidence of hyperlipidemia is extremely high in pediatric RNS; however, research on and treatment of hyperlipidemia are notably inadequate [5]. Persistent hyperlipidemia is an independent risk factor for the development and progression of glomerulosclerosis [6]. The pathogenesis of glomerulosclerosis remains unclear and the disease exhibits substantial heterogeneity. Moreover, the lack of specific therapeutic drugs, coupled with the adverse effects and uncertain outcomes associated with prolonged glucocorticoid and immunosuppressant use, impose a heavy burden on patients and their families [7]. Thus, exploring how abnormal lipid metabolism in children contributes to glomerulosclerosis in RNS may provide a theoretical basis for an effective treatments.

Focal segmental glomerulosclerosis (FSGS) is a common pathological change in pediatric INS and RNS of children [8,9]. FSGS is among the most debilitating and least treatable forms of INS and often leads to ESRD, requiring dialysis and/or transplantation [10]. Currently, treatment options for FSGS, such as immunosuppressants and renal transplantation, often yield variable results, highlighting the need for a deeper understanding of the underlying pathophysiological mechanisms that contribute to disease progression [11,12].

The dysregulation of lipid metabolism plays a pivotal role in the pathogenesis of various kidney diseases, including FSGS [13]. Alterations in lipid homeostasis can lead to cellular damage and inflammation, thereby exacerbating glomerular injury and sclerosis [14]. Specific lipid metabolic pathways may be involved in the development of glomerulosclerosis, but the precise mechanisms remain to be elucidated [15,16].

Although progress has been made in our understanding of lipid metabolism in kidney diseases, there is still a considerable gap in the identification of specific lipid-related biomarkers linked to FSGS. This gap suggests an urgent need for comprehensive research aimed at exploring the relationship between differentially expressed lipid metabolism genes and their contributions to FSGS pathogenesis. These studies may reveal potential biomarkers that can improve the diagnosis and prognosis of patients with FSGS [17].

To address this research gap, we employed a multi-faceted approach that included differential gene expression analysis, functional enrichment analysis, and protein-protein interaction (PPI) network construction. Using bioinformatics tools and experimental validation, we aimed to identify important genes related to lipid metabolism that are significantly altered in FSGS. This methodology allows systematic exploration of the role of lipid metabolism in FSGS, providing insights that could lead to the discovery of novel therapeutic targets and biomarkers for clinical use [18,19].

The primary goal of this study was to elucidate the relationship between lipid metabolism and FSGS pathophysiology. By identifying the differentially expressed genes(DEGs) involved in lipid metabolism, we hope to establish a clearer understanding of their roles in kidney function and disease progression. Our findings are expected to help develop effective diagnostic tools and targeted treatments for patients with FSGS to improve their clinical outcomes and quality of life [20].

This study aimed to advance renal pathology by offering new insights into the molecular mechanisms underlying FSGS and,highlight the importance of a deeper understanding of lipid metabolism in kidney disease.

## Materials and methods

### Data collection

Gene expression probe matrix and platform files related to FSGS were retrieved from the Gene Expression Omnibus (GEO) database (http://www.ncbi.nlm.nih.gov/geo/) based on the following criteria: (i) FSGS or focal segmental glomerulosclerosis; (ii) expression profiling by array; and (iii) *Homo sapiens*. The final datasets selected for analysis were GSE69814, GSE129973, and GSE121233. All three datasets were derived from patients with nephrotic syndrome, therefore, the heterogeneity among diseases was minimized. The GSE69814 dataset included glomerular transcriptomes from six patients diagnosed with FSGS and five patients with minimal change disease (MCD), using the GPL6244 platform (Affymetrix Human Gene 1.0 ST Array) [21]. The GSE129973 dataset contains glomerular transcriptomes from 20 kidneys affected by FSGS and corresponding unaffected tissues from 20 tumor nephrectomies, analyzed with the GPL570 platform (Affymetrix Human Transcriptome Array 2.0).The GSE121233 consists of glomerular transcriptomes from five kidneys with FSGS and the unaffected portions from five tumor nephrectomies, using the GPL17586 platform (Affymetrix Human Transcriptome Array 2.0) [22]. Additionally, 1450 lipid metabolism-related genes (LMRGs) were downloaded from three databases: the Molecular Signatures Database (MSigDB, https://www.gsea-msigdb.org/gsea/msigdb), the Kyoto Encyclopedia of Genes and Genomes (KEGG, https://www.genome.jp/kegg/), and the Genecards database (https://www.genecards.org). (S1 Table).

### Data processing

The expression matrices for the GSE69814 and GSE129973 datasets were extracted using R software (https://www.bioconductor.org/). These matrices underwent standardization and subsequent analysis via the “normalize Between Arrays” function within the “limma[3.52.2]” package of R, a method of obtaining a standardized gene expression matrix to eliminate systematic differences between different experimental conditions, technical variations, or samples..

### Identification of DEGs and differentially expressed LMRGs (DELMRGs) in FSGS

After normalization, the gene expression matrix underwent differential analysis using the “limma[3.52.2]”package in R. The “ggplot2[3.3.6]” package was used for data visualization. The criteria for statistical significance in GSE69814 were defined as a log2 fold change (FC) greater than 1.0 and a *P*-value less than 0.05. For GSE129973, the criteria were a log2 FC greater than 0.5 and a P-value less than 0.05, to ensure that possible positive results were not missed due to the small number of included values. A volcano plot of DEGs was created using the “ggplot2[3.3.6]” package. The intersection of LMRGs with DEGs was analyzed using both “ggplot2[3.3.6]” and “VennDiagram[1.7.3]” packages to identify differentially expressed LMRGs (DELMRGs), resulting in the identification of 56 DELMRGs.

### Go annotation(GO) and KEGG pathway enrichment analysis

Subsequently, GO annotation and KEGG pathway enrichment analyses were executed using the “ggplot2[3.3.6]” package in R. GO analysis included biological processes (BPs), cellular components (CCs), and molecular functions (MFs). The thresholds for the enrichment analysis results were set at normalized enrichment scores (|NES|>1) and adjusted p-values of 0.05.

### PPI network construction and hub genes

The STRING database (https://cn.string-db.org/) was used for information on known and predicted protein interactions. A confidence score of 0.4 was established to denote medium confidence in the interaction data between the DELMRGs. PPI networks were visualized using the Cytoscape software [3.10.2], Which is a widely used software platform for visualizing and analyzing biological network data. The CytoHubba plug-in, which utilizes five core algorithms (MCC/MNC/Degree/EPC/Closeness), facilitated the identification of hub genes, resulting in the selection of five hub DELMRGs.

### Receiver operating characteristics (ROC) Curve and expression matrix analysis

The diagnostic accuracy of these genes was further assessed using ROC curve analysis, which included the calculation of the area under the ROC curve(AUC). We analyzed the expression patterns of the five hub DELMRGs in the GSE121233 dataset to evaluate the generalizability of the identified biomarkers, which served as the training set. Data processing methods removed samples missing from either variable. ROC curves were generated using the pROC package [1.18.0] and ggplot2 [3.3.6], with successful prediction performance indicated by an AUC >  0.7. We also analyzed the expression differences of the five hub DELMRGs in the GSE121233 dataset.

### Immunohistochemical (IHC) verification

To validate the five hub DELMRGs, IHC analysis was conducted across three groups: normal renal tissue (uninfected paracarcinoma kidney tissue located > 3 cm from the tumor site following nephrectomy for nephroblastoma), MCD, and FSGS. IHC verification involved tissue samples from six patients in each cohort. The tissue sections were incubated at 60 °C for 12 h, dewaxed in xylene, and hydrated using a graded alcohol series. The slides were placed in Tris-EDTA buffer (pH 9.0) in a microwave oven for antigen repair. Endogenous peroxidase blockers were used to eliminate endogenous interference and normal goat serum was used to block nonspecific antigens at room temperature. Five polyclonal antibodies (Abcam, Shanghai, China) were used to probe the slices at 4 °C overnight. The sections were then incubated with a secondary antibody (Servicebio, Wuhan, China) at 23 °C for 30 min. The samples were then stained with diaminobenzidine, counterstained with hematoxylin, and dehydrated using an alcohol gradient. The IHC staining results were independently assessed by two observers who were blinded to the patients’ clinical information. The evaluation methods included the random selection of five glomerular visual fields from each sample for imaging, with the integrated optical density (IOD) values and glomerular areas quantified using Image J [1.54g]. We computed the average optical density as the IOD divided by the glomerular area, which quantitatively measured the expression levels of the five hub DELMRGs in the glomerular region. The final value for each sample was derived by averaging the optical density measurements from the five fields. All pathological specimens were obtained from patients hospitalized at the First Affiliated Hospital of Guangxi Medical University between January 2020 and June 2024, with the collection date being August 1, 2024. Kidney tissue samples collected from the patients involved in this study were obtained in strict adherence to medical ethics guidelines and approved by the Ethics Committee of the First Affiliated Hospital of Guangxi Medical University (Ethics number: 2024-K273-01)

### Correlation analysis between ECHS1 and clinical observation indicators

Through clinical correlation analysis of ECHS1, we hoped to elucidate its function under specific physiological and pathological conditions, especially renal function and lipid-related indicators, to further explore its feasibility as a therapeutic target. we expanded the sample size of immunohistochemical tissues. We selected several clinical observation indicators, including albumin (ALB, g/L), creatinine (CRE, µmol/L), blood urea nitrogen (BUN, µmol/L), creatinine clearance rate (CCR, mL/min), cystatin C (CYSC, mg/L), total cholesterol (TCHO, mmol/L), triglycerides (TG, mmol/L), high-density lipoprotein (HDL, mmol/L), low-density lipoprotein (LDL, mmol/L), non-high-density lipoprotein (non-HDL, mmol/L), and 24-hour urinary protein (24hU-Pr,mg/24h). Owing to the absence of many clinical observational indicators in the normal group, particularly the lack of lipid-related data in some patients and the near-total absence of 24hU-Pr data, we excluded the normal group from subsequent evaluations. In our study of the correlation between ECHS1 and clinical observation indicators, we increased the sample size to 30 patients (15 in the MCD group and 15 in the FSGS group). The results of the last examination before renal puncture were used as inclusion criteria, and the use of lipid-regulating drugs and dialysis treatment was used as exclusion criteria. Spearman’s correlation was used to analyze the relationships among a limited number of clinical samples. All clinical case data were sourced from patients hospitalized at the First Affiliated Hospital of Guangxi Medical University between January 2020 and June 2024, with the data collection date being August 1, 2024.

### Statistical Analysis

Statistical analyses were performed using the stats [4.2.1] and car [3.1-0] packages in the R software, and data visualization via ggplot2 [3.4.4]. We used an independent sample T test to analyze the expression differences of the five hub DELMRGs in the GSE121233 dataset. We used the Kruskal–Wallis and Dunn’s test to analyze the expression differences of the five hub DELMRGs in different IHC tissue samples. We used the Wilcoxon rank sum test to analyze the differences in *ECHS1* expression in expanded tissue samples. Statistical significance was set as *P* <  0.05 indicated expression differences between groups

## Results

### DEGs of the GSE69814 and GSE129973 dataset

**Fig 1:**
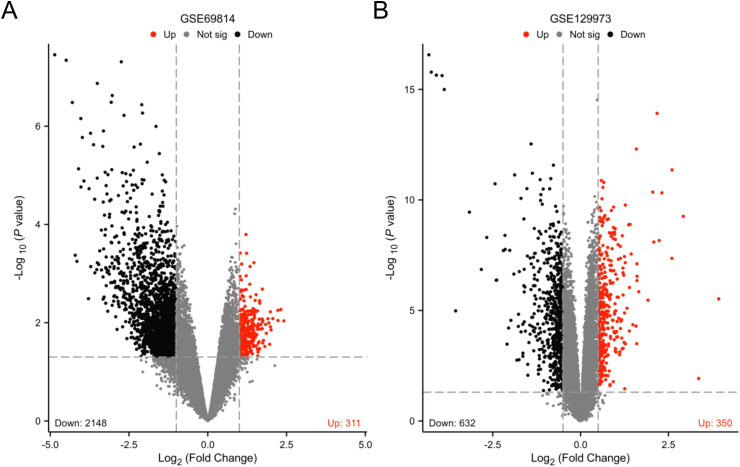
Differentially expressed genes of FSGS (A) Volcano plot for the DEGs identified from GSE69814. (B) Volcano plot for the DEGs identified from GSE129973. Red and blue plot triangles represent DEGs with up-regulated and down-regulated gene expression, respectively.DEG, differentially expressed gene; FSGS, focal segmental glomerulosclerosis.

The batch effect was removed from the GSE69814 and GSE129973 dataset, and screening criteria for GSE69814 were log2FC > 1.0 and *P* < 0.05 for significant differences. The screening criteria for GSE129973 were log2FC > 0.5 and P < 0.05 for significant differences. Subsequently,2459 DEGs were finally obtained from the GSE69814 dataset including 311 upregulated and 2148 downregulated genes.(Fig 1A and S1 Table). Similarly, in the GSE129973 dataset, we identified 982 DEGs, which included 350 upregulated and 632 downregulated genes (Fig 1B and S1 Table).

### A total of 56 genes were determined to be connected to FSGS and lipid metabolism

**Fig 2:**
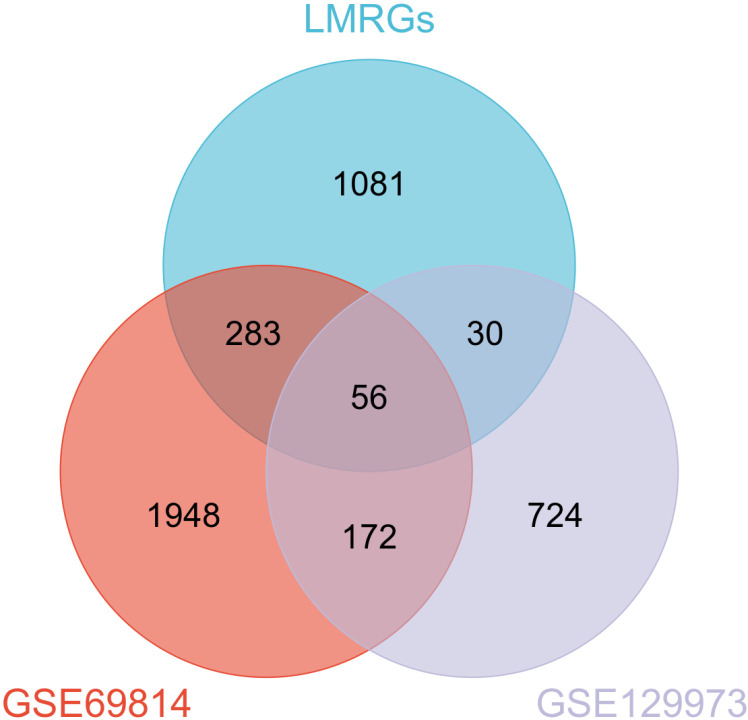
Venn diagram of FSGS DEGs from GSE69814, DEGs from GSE129973 and LMRGs; Blue represents LMRGs, orange represents FSGS DEGs from GSE69814 and mauve represents FSGS DEGs from GSE129973. DEG, differentially expressed gene; FSGS, focal segmental glomerulosclerosis; LMRGs, lipid metabolism-related genes.

Subsequently, 2,459 DEGs from the GSE69814 dataset, 982 DEGs from the GSE129973 dataset, and 1,450 LMRGs were analyzed to establish the intersection, yielding 56 DELMRGs associated with FSGS (Fig 2 and S1 Table).

### GO annotation and KEGG pathway enrichment analysis of DELMRGs in FSGS

**Fig 3:**
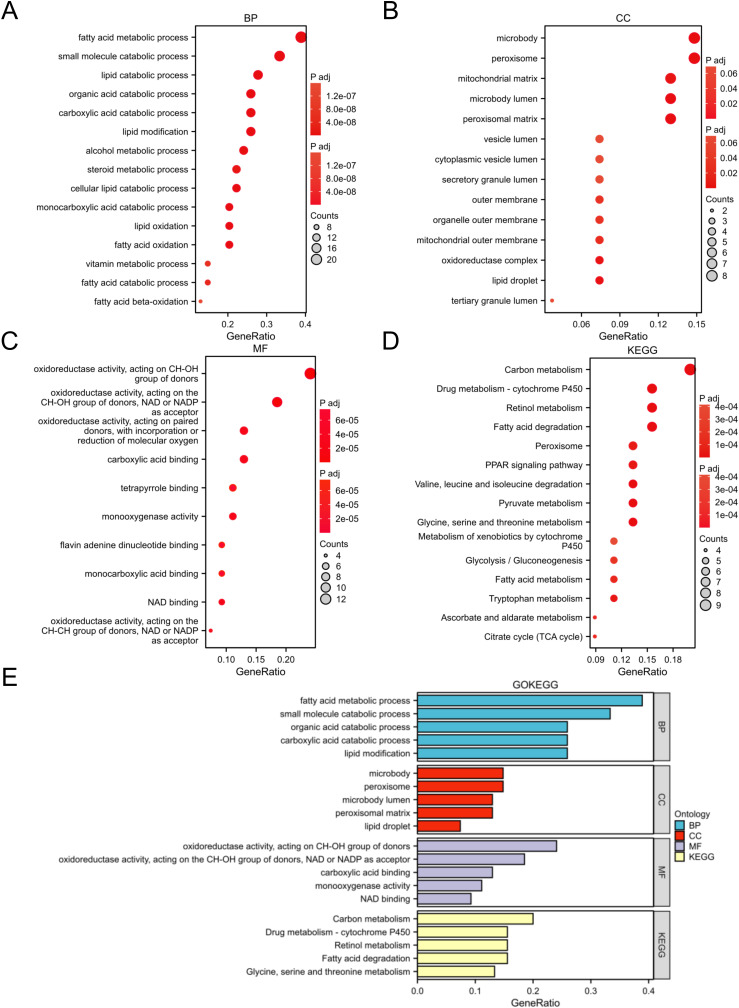
Enrichment analysis of DELMRGs. **A** BP analysis of the DELMRGs. **B** CC analysis of the DELMRGs. **C** MF analysis of the DELMRGs. **D** KEGG pathway analysis of the DELMRGs. **E** Top five GO and KEGG analysis in every category of the DELMRGs.BP, biological process; CC, cellular component; MF, molecular function; KEGG, Kyoto Encyclopedia of Genes and Genomes; DELMRG, differentially expressed lipid metabolism-related gene.

Enrichment analysis was performed on the identified DELMRGs to explore their BPs and signaling pathways. The BP analysis of GO annotation showed these DELMRGs mainly involved in the terms of “fatty acid metabolic process,”,“small molecule catabolic process”, and “lipid catabolic process” (Fig 3A), mainly related to lipid metabolism. The CC analysis of GO annotations illustrated that the proteins expressed by these DELMRGs were mainly localized in “microbody,” “peroxisome”, and “mitochondrial matrix” (Fig 3B), mainly organelles involved in lipid metabolism. The MF analysis of GO annotation showed that these DELMRGs primarily perform functions,such as “oxidoreductase activity on CH-OH groups of donors,” “oxidoreductase activity using NAD or NADP as acceptors,” and “oxidoreductase activity involving paired donors with the incorporation or reduction of molecular oxygen” (Fig 3C), mainly related to fatty acid β oxidation and emergency oxidation-related processes. The KEGG pathway enrichment analysis revealed that the identified DELMRGs were primarily associated with signaling pathways, such as “carbon metabolism,” “drug metabolism-cytochrome P450,” and “retinol metabolism” (Fig 3D). The top five processes ranked according to BP, CC, MF and KEGG are shown in Fig 3E.

### Construction of the PPI network and identification of hub DELMRGs

**Fig 4:**
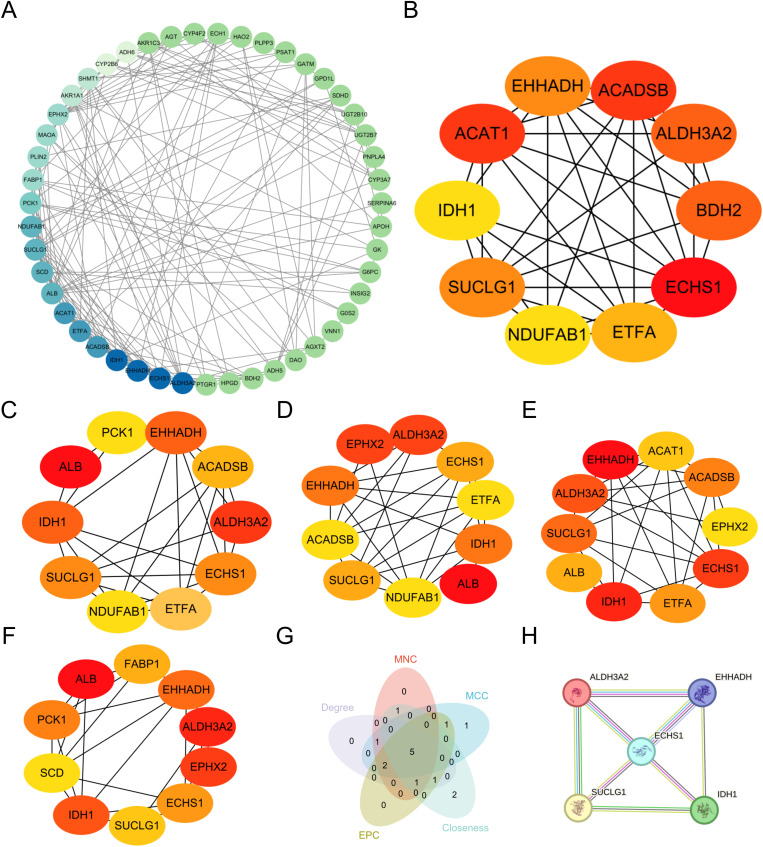
PPI network of DELMRGs and hub DELMRGs. **A** PPI network of 56 DELMRGs;from blue to green, the darker the color, the more nodes are connected. **B–F** Top 10 hub DELMRGs by five algorithms (MCC, MNC, Degree, EPC, and Closeness);the darker the color, the higher the ranking. **G** Venn diagram of five algorithms**. H** The five hub DELMRGs.DELMRG, differentially expressed lipid metabolism-related gene; PPI, protein-protein interaction.

PPI analysis was conducted using the STRING database to explore the interactions among proteins expressed by the identified DELMRGs. The resulting PPI network, constructed using Cytoscape software, comprised 56 nodes and 171 edges (Fig 4A). Five algorithms (MCC, MNC, Degree, EPC, and Closeness) were used to predict the top 10 hub DELMRGs (Fig 4B–4F and S2 Table). Ultimately, five hub DELMRGs (*ECHS1, EHHADH, IDH1, SUCLG1and ALDH3A2*) consistently ranked within the top 10 across all five prediction algorithms and were thus classified as hub DELMRGs (Fig 4G–4H).

### Diagnostic ROC curves and expression levels of five hub DELMRGs

**Fig 5:**
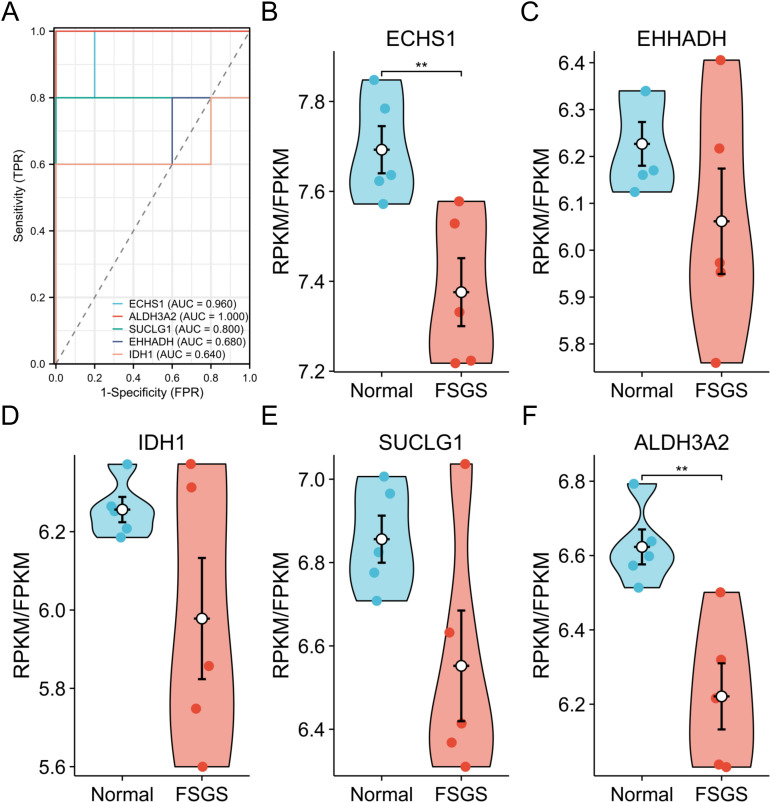
Diagnostic ROC curves and expression levels of five hub DELMRGs. **A** Diagnostic ROC curves of 5 hub DELMRGs in GSE121233 dataset. **B-F** Expression levels of 5 hub DELMRGs (*ECHS1, EHHADH, IDH1, SUCLG1*, and *ALDH3A2*) in GSE121233 dataset. **P means *P* < 0.01.ROC, receiver operating characteristic; DELMRG, differentially expressed lipid metabolism-related gene.

To assess the diagnostic capabilities of these five hub DELMRGs, we used the AUC derived from the ROC analysis conducted on the GSE121233 dataset, which was designated as the training set (Fig 5A). In this dataset, *ECHS1*, *ALDH3A2* and *SUCLG1* showed relatively good diagnostic value. The AUC for *EHHADH* and *ECHS1* was greater than 0.95, indicating a strong diagnostic potential. Using the original gene expression profile data from this dataset, we validated the expression trends of the five hub DELMRGs (Fig 5B–5F). Furthermore, we observed statistically significant differences in the expression levels of *ECHS1* and *ALDH3A2* between the two groups of glomerular samples.

### Only one DELMRG, *ECHS1*, was identified using IHC

**Fig 6:**
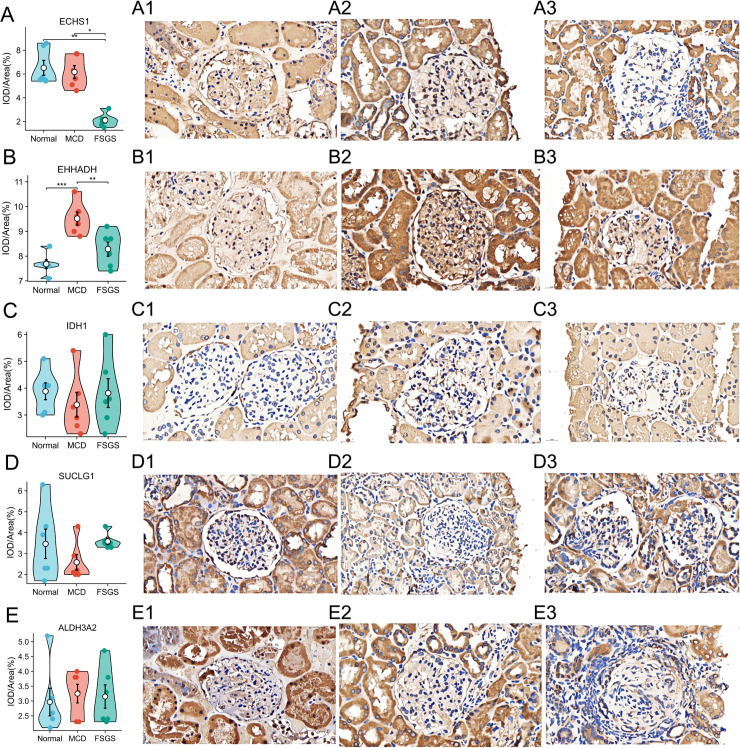
Expression levels of 5 hub DELMRGs in our clinicopathological specimens using IHC. **A–E**. Expression levels of five hub DELMRGs (*ECHS1, EHHADH, IDH1, SUCLG1,* and *ALDH3A2*) in Normal, MCD and FSGS groups. **A1–E1** IHC photos of five hub DELMRGs (*ECHS1, EHHADH, IDH1, SUCLG1,* and *ALDH3A2*) in Normal group. **A2–E2** IHC photos of five hub DELMRGs (*ECHS1, EHHADH, IDH1, SUCLG1,* and *ALDH3A2*) in MCD group. **A3–E3** IHC photos of five hub DELMRGs (*ECHS1, EHHADH, IDH1, SUCLG1,* and *ALDH3A2*) in FSGS group. *  *P* < 0.05; **P < 0.01; *** *P* < 0.001..DELMRG, differentially expressed lipid metabolism-related gene; IHC, immunohistochemical; MCD, minimal change disease; FSGS, focal segmental glomerulosclerosis.

Although the five hub DELMRGs in the GSE121233 dataset showed different predictive values and expression levels, they were included in the following IHC validation to ensure comprehensive data collection. To further establish the clinical predictive significance of the five hub DELMRGs, we performed IHC validation using our clinicopathological specimens. These specimens, including renal tissues from children diagnosed with FSGS and MCD, were collected from pediatric patients with RNS. Normal specimens were obtained from paracarcinoma kidney tissues located over 3 cm from the tumor site post-nephrectomy for nephroblastoma in children. All pediatric patients were admitted to the Department of Pediatrics or Urology at the First Affiliated Hospital of Guangxi Medical University between 2020 and 2024, and the specimens were preserved in the Department of Pathology of the hospital. Ultimately, *ECHS1* was the only gene that showed significant differences in expression between the Normal and the FSGS groups, as well as between the MCD and the FSGS groups (Fig 6 and S3Table).

### Correlation analysis between ECHS1 and clinical observation indicators

**Fig 7:**
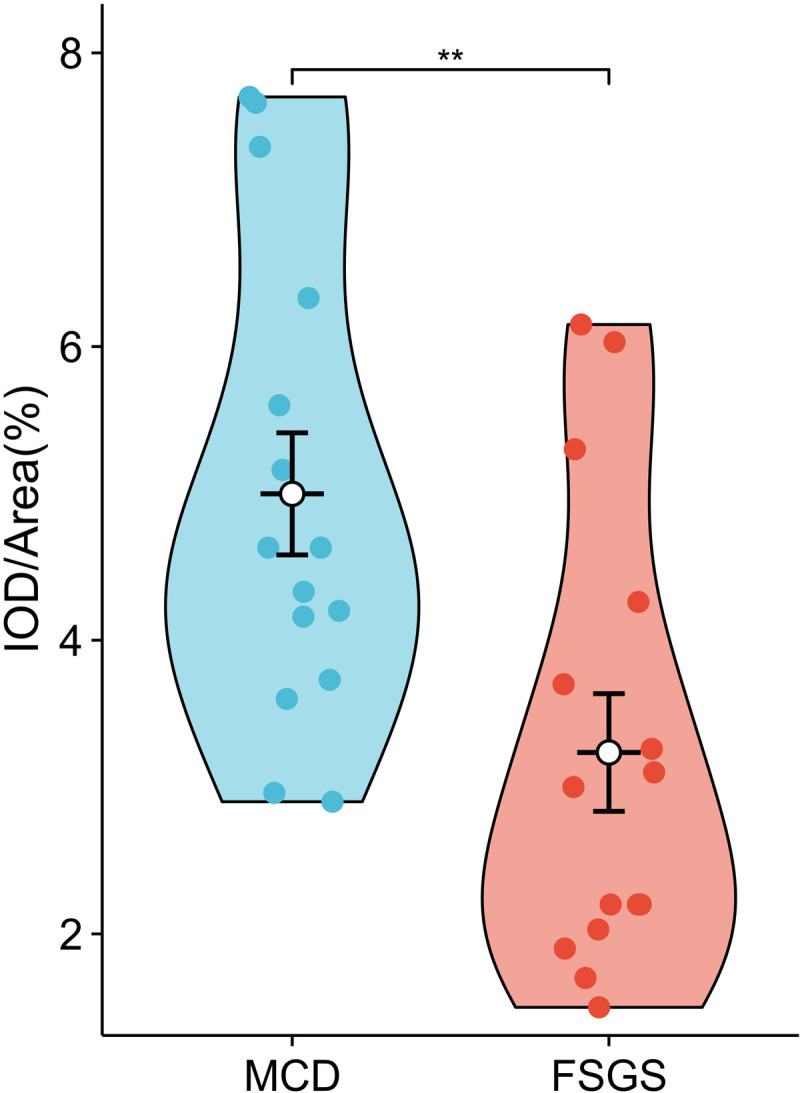
Expression levels of ECHS1 in MCD and FSGS group. **P < 0.01. FSGS, focal segmental glomerulosclerosis; MCD, minimal change disease; FSGS, focal segmental glomerulosclerosis.

Expansion of samples from the MCD and FSGS groups revealed a more significant difference in *ECHS1* expression between the two groups (Fig 7 and S4 Table).In our small sample, we found no significant correlation between *ECHS1* expression and clinical observation indicators, including ALB, CRE, BUN, CCR, CYSC, TCHO, TG, HDL,LDL, non-HDL and 24hU-Pr. (S4 and S5 Tables).

## Discussion

FSGS is a significant and progressive kidney disease characterized by glomerular scarring, which can lead to renal failure and increased mortality rates among affected individuals [23]. This disease substantially burdens healthcare systems because of its complex management and the high costs of treatments, such as dialysis and transplantation [24].Current treatment strategies, such as medication and lifestyle changes, often have limited effectiveness and vary widely in their response among patients. This highlights the urgent need for new biomarkers and therapeutic targets to improve early diagnosis and treatment of this debilitating condition [25,26].

By employing comprehensive bioinformatics analyses, we identified 56 DELMRGs, among which *ECHS1* emerged as a key player in the disease mechanism. This investigation not only sheds light on the potential role of lipid metabolism in FSGS but also provides a foundation for future research into targeted therapies and early diagnostic tools that may ultimately improve patient outcomes [27]. Our findings revealed a significant association between DELMRGs and FSGS pathogenesis. Specifically, the decreased expression of *ECHS1* suggests a potential disruption in lipid metabolic pathways that could contribute to disease progression. Previous studies have indicated that lipid accumulation and altered metabolism can exacerbate renal injury and fibrosis, highlighting the vital role that lipid homeostasis plays in maintaining glomerular health [14,28,29].

Pathway enrichment analysis further supported our findings, revealing that the identified DEGs were significantly associated with important lipid metabolic pathways, including fatty acid and cholesterol metabolisms. KEGG analysis demonstrated notable enrichment of these pathways, suggesting that lipid dysregulation could be contribute to the development of FSGS. This is consistent with the existing literature highlighting the harmful effects of dyslipidemia on kidney function, where lipid metabolism abnormalities contribute to the progression of renal diseases [30]. Future studies should aim to clarify how these lipid metabolic pathways affect FSGS pathophysiology to identifyl new therapeutic targets [5]. Targeting these pathways may mitigate disease progression and improve patient outcomes, emphasizing the importance of lipid metabolism in renal pathology [31,32].

Construction of a PPI network, which identified five hub genes among the DELMRGs, provided additional insights into the molecular mechanisms underlying FSGS. *ECHS1*, a hub DELMRG, has been previously implicated in mitochondrial function, which is consistent with its observed enrichment in processes, such as fatty acid metabolism. Similar to *ECHS1*, *EHHADH* is a key enzyme in the fatty acid β-oxidation [33]. *IDH1, ALDH3A2, SUCLG1*, three other hub genes are also primarily enriched in the bypass of fatty acid β-oxidation [34–36]. The high connectivity between these hub genes suggests that they play crucial roles in the biological framework of the disease. The identification of these key regulatory nodes was consistent with findings from other studies that have linked specific proteins to renal disease mechanisms, including cell apoptosis and fibrosis [37]. Moreover, understanding the interactions between these hub genes could help develop targeted therapies to restore lipid balance and prevent renal injury. This approach could be particularly beneficial in patients with FSGS for whom traditional treatments have shown limited efficacy [38].

Validation of our findings through IHC confirmed that only *ECHS1* was significantly expressed in FSGS tissues compared with that in normal and MCD tissues. This supports the transcriptomic data and highlights the potential of *ECHS1* as a reliable biomarker of FSGS. Reduced *ECHS1* expression in renal pathology indicates that it may be an indicator of disease severity and progression. Previous studies have indicated that alterations in the expression of LMRGs are correlated with the extent of renal damage, further supporting their role as biomarkers in clinical settings [39]. When the clinicopathological classification of the kidney is difficult to determine, *ECHS1* may be helpful in classifying it. Therefore, *ECHS1* may be a potential therapeutic target for glomerular sclerosis and renal fibrosis.

ECHS1 is an enzyme that plays a pivotal role in organisms [40]. This enzyme primarily participates in the second step of fatty acid β-oxidation, where it catalyzes the hydration of 2-trans-enoyl-CoA to form 3-hydroxyacyl-CoA [41]. This step represents a critical juncture in the fatty acid β-oxidation process and is important for maintaining the energy metabolic balance in organisms [41]. ECHS1 is encoded by the *ECHS1* gene and is expressed in various organs and tissues throughout the human body, with particularly prominent expression in the liver, kidney, and adipocytes [42]. Early research on ECHS1 focused on congenital metabolic diseases, such as Leigh syndrome [41,43]. Subsequent studies revealed that ECHS1 exerts oncogenic or tumor-suppressive effects by mediating metabolic reprogramming in multiple cancers, as evidenced by its aberrant expression [42,44,45]. ECHS1 may protect against myocardial damage caused by doxorubicin [46].However, no study has investigated the correlation between ECHS1 and glomerular sclerosis. Future studies should explore the functional role of ECHS1 in renal cells to elucidate its contribution to glomerular integrity and function, potentially paving the way for innovative therapeutic interventions.

The datasets for this study were derived from adult patient samples; however, we validated it using pediatric patient samples. Same of the results were similar, but most were significantly different. This result also suggests that some pathophysiological processes in children differ in adults [47,48]. Specifically, FSGS in children are usually caused by primary diseases [49], and the pathogenesis may be more directly related to genetic mutations or specific immune responses [50]. However, the pathogenesis of adult FSGS may be more directly related to the inflammatory response and influence of secondary factors is more substantial [51]. Future clinical studies should consider these differences [52].

No significant correlation was found between *ECHS1* expression and clinical process measurements in our small-sample analysis. However, after we expanded the tissue sample size, *ECHS1* was still expressed at significantly different levels in kidney tissues with different pathologies. Diseased kidneys often do not have safe blood lipid levels [53,54]. Lipid nephrotoxicity can also occur in diseased kidneys in the presence of normal lipids [55]. Therefore, more attention should be paid to lipid nephrotoxicity in kidney disease.

This study has some limitations.. First, the relatively small sample size may restrict the generalizability of our findings; a larger cohort would provide more robust statistical power and enhance the reliability of the identified biomarkers. Additionally, the absence of basic laboratory experiments limits the depth of mechanistic insights that could be drawn from our results, as gene expression alone does not elucidate functional pathways. Furthermore, potential batch effects among the datasets used for analysis could introduce variability, thereby influencing the observed associations. These limitations underscore the necessity for further validation with larger studies and complementary experimental methods to confirm our findings and their clinical relevance in pediatric FSGS.

## Conclusion

This studies elucidats the significant role of DELMRGs in FSGS pathogenesis, particularly emphasizing the potential biomarker function of *ECHS1*.This gene has the potential to enhance early diagnosis and therapeutic strategies. By combining bioinformatics with experimental validation, we provided insights into the molecular mechanisms underlying renal pathologies, ultimately improving patient management and treatment outcomes in clinical practice.

## Supporting information

S1 Table Lists of LMRGs,DEGs and DELMRGs. (XLSX)

S2 Table lists of 5 algorithms. (XLSX)

S3 Table Glomerulus IOD/Glomerulus Area(%). (XLSX)

S4 Table ECHS1 expression level of patients. (XLSX)

S5 Table ECHS1 and clinical observation indicators. (DOCX)
