# Supplementary material for: ECHS1 as a Lipid Metabolism Biomarker for Pediatric Focal Segmental Glomerulosclerosis
Source: PLoS One. 2025 Mar 10;20(3):e0319049. doi: 10.1371/journal.pone.0319049 (PMC11893130; doi:10.1371/journal.pone.0319049)
Supplement: S5 Table — (DOCX) [file pone.0319049.s005.docx]

**S5 Table.** **ECHS1 and clinical observation indicators**

| clinical observation indicators | *r* | *P* |
| --- | --- | --- |
| ALB（g/l）  BUN（mmol/l）  CRE（umol/l）  CCR（ml/min）  CYSC（mg/l）  TCHO（mmol/l）  TG（mmol/l）  HDL（mmol/l）  LDL（mmol/l）  non-HDL（mmol/l）  24hU-Pr（mg/24h） | -0.175  0.204  0.173  -0.056  0.030  0.100  0.043  -0.018  -0.176  -0.043  0.225 | 0.354  0.279  0.361  0.770  0.877  0.599  0.825  0.927  0.353  0.820  0.231 |
